# Comprehensive transcriptional landscape of aging mouse liver

**DOI:** 10.1186/s12864-015-2061-8

**Published:** 2015-11-05

**Authors:** Ryan R. White, Brandon Milholland, Sheila L. MacRae, Mingyan Lin, Deyou Zheng, Jan Vijg

**Affiliations:** Department of Genetics, Albert Einstein College of Medicine, Bronx, NY USA; Department of Neuroscience, Albert Einstein College of Medicine, Bronx, NY USA; Albert Einstein College of Medicine, Michael F. Price Center for Genetic and Translational Research, 1301 Morris Park Ave, Bronx, NY 10461 USA

**Keywords:** Gene expression, Aging, RNA-seq, Transcriptome, Non-coding RNA, Variation, Liver

## Abstract

**Background:**

Mammalian aging is a highly complex process, a full mechanistic understanding of which is still lacking. One way to help understand the molecular changes underlying aging is through a comprehensive analysis of the transcriptome, the primary determinant of age-related phenotypic diversity. Previous studies have relied on microarray analysis to examine gene expression profiles in different tissues of aging organisms. However, studies have shown microarray-based transcriptional profiling is less accurate and not fully capable of capturing certain intricacies of the global transcriptome.

**Methods:**

Here, using directional whole transcriptome RNA-sequencing of aged mouse liver we have identified a comprehensive high-resolution profile of differentially expressed liver transcripts comprised of canonical protein-coding transcripts, transcript isoforms, and non-coding RNA transcripts, including pseudogenes, long non-coding RNAs and small RNA species.

**Results:**

Results show extensive age-related changes in every component of the mouse liver transcriptome and a pronounced increase in inter-individual variation. Functional annotation of the protein-coding mRNAs and isoforms indicated broad alterations in immune response, cell activation, metabolic processes, and RNA modification. Interestingly, multiple lncRNAs (*Meg3*, *Rian*, *Mirg*) from the *Dlk-Dio3* microRNA locus were found up-regulated in aging liver, classifying this locus as a putative regulatory hotspot locus in aging liver. Moreover, integration of the altered non-coding RNAs and protein-coding transcripts into interaction networks of age-related change revealed inflammation, cellular proliferation, and metabolism as the dominant aging phenotypes in mouse liver.

**Conclusions:**

Our analyses provide the first comprehensive dissection of the transcriptional landscape in aging mouse liver.

**Electronic supplementary material:**

The online version of this article (doi:10.1186/s12864-015-2061-8) contains supplementary material, which is available to authorized users.

## Background

Mammalian aging is a complex biological process that still remains poorly understood. To increase our understanding of the different interacting processes that underlie age-related organ and tissue degeneration, a systematic study of alterations in gene expression is a logical starting point. Global changes in gene expression are a “hallmark” of aging in multiple species [1, 2] and have been successfully used to demonstrate specific effects of interventions that slow aging, such as caloric restriction [3]. In the past, microarray analyses have been used extensively to study age-related differences across multiple tissues, but ultimately these analyses proved insufficient for gaining a complete understanding of the aging transcriptome for various reasons. First, microarray analysis relies on fully annotated genes and since the release of the ENCODE project, we now know that novel un-annotated transcripts are constantly being discovered [4]. Second, microarray analyses generally suffer from excessive noise, which could explain the very small overlap between different published results of gene expression changes with age in mouse liver [5]. For example, while one report mentioned minimal age-related changes in gene expression during mouse liver aging [6], others observed a considerable number — albeit highly variable from study to study — of such changes [5, 7–9]. Finally, microarray analysis is generally not capable of detecting altered isoform levels, due to differential splicing, or changes in non-mRNA species, such as non-coding (nc)RNAs. This is especially important for non-coding RNAs since by now the role of this category of transcripts is generally understood to involve fine-tuning gene regulatory patterns, such as cellular metabolism [10]. Increased deregulation at this level could potentially explain many of the aging phenotypes observed, which involve subtle rather than dramatic changes. Recent advances in RNA sequencing now offer a suitable alternative to studying variation in gene expression that addresses all of these issues.

While changes in gene expression profiles have been used as biomarkers for aging and sometimes even as predictors of biological aging rate [8], the ultimate aim in studying the aging transcriptome is to elucidate the pathways that define age-related degenerative processes and the responses they evoke, in a tissue-specific manner. For this purpose it is essential to collect mRNA expression data in the context of their non-mRNA, gene regulatory RNAs. Indeed, as mentioned, apart from the canonical protein-coding mRNAs, which make up about 3 % of the genome, the critical role of a multitude of ncRNAs are now well recognized. NcRNA is a broad umbrella term that includes pseudogenes, or those genes that have lost their protein-coding potential but are sometimes still capable of being transcribed [11], long non-coding RNAs (lncRNAs), typically >200 nucleotides long, which have been shown to perform a wide range of functions from transcriptional de-repression to silencing [12], and small ncRNAs (<200 nucleotides in length), which are mainly comprise the regulatory microRNAs (miRNAs), small nuclear RNAs (snRNAs), small nucleolar RNAs (snoRNAs), and transfer RNAs (tRNAs). Although species in this last category are small, they may have a large impact on cellular function, regulating such processes as gene silencing, splicing, and translation [13]. The role of ncRNAs in aging remains unclear, which essentially constrains attempts to generate comprehensive functional networks of tissue-specific alterations in the RNA landscape.

Here, we utilized an RNA-sequencing approach capable of capturing the whole transcriptome of mouse liver, a similar approach that has been performed in aging brain for other mammals [14, 15]. Specifically, we analyzed young adult (4 months) and old (28 months) mouse livers and identified significantly altered transcripts, not only canonical coding transcripts, but also novel ncRNA transcripts and isoform variants. Several of the altered ncRNAs, many of them from the *Dlk*-*Dio3* locus — an imprinted domain implicated in development and pluripotency and here identified as a ncRNA gene regulatory hotpot in mouse liver aging — are putative regulators of the age-related loss of mitotic potential. Age-related differentially expressed transcripts, including isoforms and ncRNAs, were first compiled into putative functional pathways and then used for constructing age-related interaction networks. These networks of age-related change revealed three dominant, emerging phenotypes: inflammation, proliferative homeostasis, and lipid metabolic changes.

## Results

### Sequencing metrics and transcriptome genomic coverage

To directly analyze the global transcriptome of aging mouse liver, we isolated total RNA from three young adult (4 months) and three old (28 months) male mouse livers and performed directional whole transcriptome sequencing [16]. For each library we generated an average of ~30 million paired-end reads of which 82–90 % could be mapped to the NCBI Build 37/mm9 reference genome (Additional file 1: Table S1). Aligned reads were characterized using a unique annotation reference database that combined five different known databases for all transcripts: ENSEMBL, GENBANK/NCBI, REFSEQ, VEGA/HAVANA, and MGI. By taking this approach we were able to take advantage of all known database predictions and compile these for novel transcript discovery in aging [17]. Thus, we were able to analyze not only manually curated gene sets, as is the case with VEGA/HAVANA annotations, but also parts of the mouse genome that have only had computationally predicted annotation, such as those found in ENSEMBL, for a total of 47,510 transcript annotations (as compared to ~35,000 for current microarray chips).

**Fig. 1:**
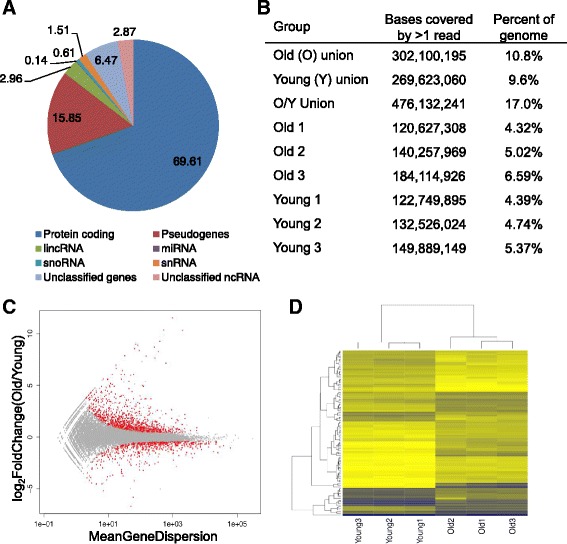
Global expression analysis in aging mouse liver. **a** Percent of globally expressed transcript subspecies expressed across both young and old livers from our unique annotation set of 47,510 annotated transcripts. **b** Table represents the number of genomic bases covered by at least one unique read in either all old livers, all young livers, or both old and young livers. Total mouse genome size reference used at time of analysis using GRCm38 build is 2,293,712,140 bases for a haploid genome. **c** Differential transcript expression plotted log_2_FoldChange(Old/Young) versus the mean gene dispersion value. Red points represent all 1264 significant differentially expressed transcripts with FDR <0.1. Old (*n* = 3), Young (*n* = 3). **d** Heatmap representation of the top 100 differentially expressed transcripts by *P*-value, generated using unsupervised hierarchical clustering

To begin, we determined the percentage of the genome actively transcribed. On average 59.5 % of the annotated transcripts from our unique annotation database of 47,510 transcripts were found to be expressed in liver of all animals combined, both young and old, as defined by >1 count per transcript to account for low-abundance transcripts. Of these transcripts ~70 % were protein-coding genes (Fig. 1a), with transcripts from pseudogenes comprising ~16 % and the remaining ~7.5 % representing ncRNAs, such as lncRNAs, snoRNAs, snRNAs, and miRNAs. About 3 % of these ncRNAs still remain unclassified (Fig. 1a). These results corroborate findings by others for the human genome [18].

In view of some long-standing hypotheses that the repression of genes normally not expressed in a differentiated tissue is relaxed at old age [19], we compared transcriptome genomic coverage between young and old livers. Of note, to fully capture the complete transcriptome from a single data set could require well over a billion RNA-seq reads rather than the roughly 150 million for all six of our animals combined. Nevertheless, we calculated the number of unique bases covered by at least one read for each animal, either young or old, and divided this by the total number of bases in the haploid mouse genome, ~2.8 billion bp [18]. The results indicate no significant difference between young and old mouse liver (Fig. 1b). Hence, we did not observe any obvious major relaxation of gene repression at old age, albeit some minor effect cannot be ruled out. Indeed, when we combined the three individual liver samples for each age group, genome coverage doubled, i.e., from ~4.8 and ~5.3 % to 9.6 and 10.8 % for the young and old group, respectively, indicating that we are far from capturing all transcribed bases in one animal. When combining data from all animals, both young and old, coverage did not double but increased only to 17.0 %, suggesting the beginning of some saturation (Fig. 1b). Nonetheless, it is still striking that up to 17 % of the mouse liver genome is capable of being transcribed.

### Aging is characterized by increased individual variation in the liver transcriptome

Increased variation of gene expression has been considered one mechanism by which organisms undergo age-related cellular degeneration [20, 21]. While we only have RNA-seq data on three young and three old animals, for each animal well over 20 million mappable reads were obtained. With high transcriptome coverage per individual animal we should, in theory, be able to accurately detect variation between animals.

**Fig. 2:**
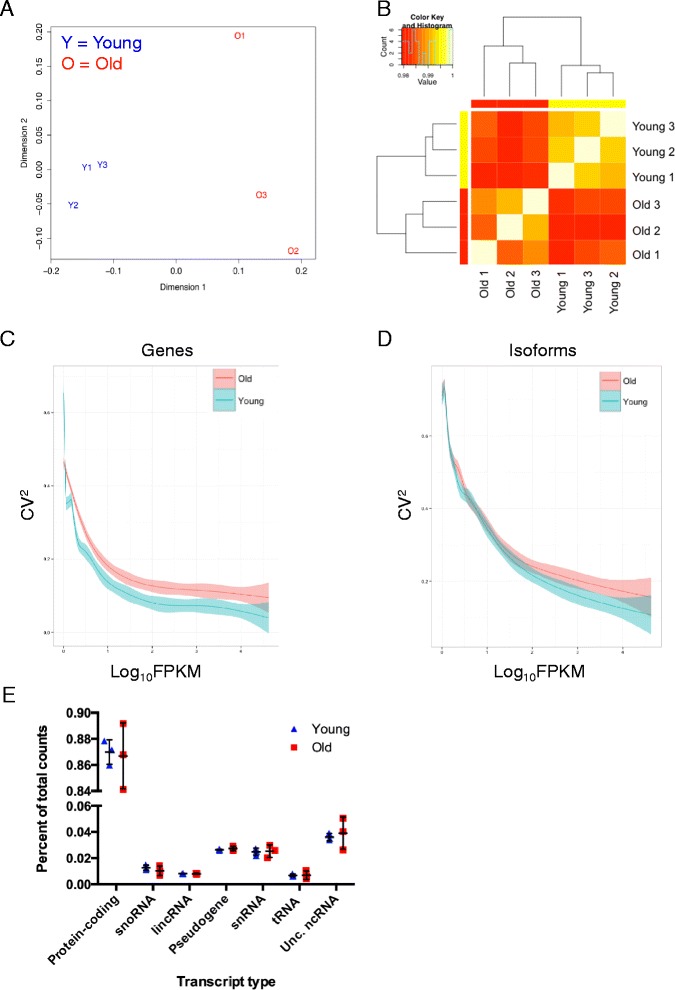
Aging profiles characterized by stochastic variation. **a** Variance of the top 25,000 transcripts plotted using multi-dimensional scaling analysis; *Y* young, *O* old, *n* biological replicate. **b** Heatmap showing Euclidean distance matrix between young and old replicate samples calculated from the variance in count values where values closer to 1 are less variable. **c, d** The calculated mean squared coefficient of variation (CV^2^) for genes (**b**) and isoforms (**d**) plotted as a function of normalized count expression for young and old liver samples. **e** The percentage of normalized individual count data for each transcript type. Black bars represent the mean ± standard deviation

To compare animal-to-animal variation at young and old age we generated a multiple dimensional scaling plot (MDS) of the variance of the top 25,000 expressed transcripts (Fig. 2a). MDS analysis shows that young animals cluster together, while old individuals have a clear high degree of variance between each biological replicate. Furthermore, we analyzed each sample using unsupervised Euclidean matrix plots to observe the variance between the individual young and old livers (Fig. 2b). We find that young and old still separate to each respective class based on variance alone. Moreover, we find that young compared to young liver has a distance value closer to one, while old compared to old liver have a value further from one, meaning aged liver transcriptomes are more individually variable than their younger counterpart.

When analyzing the variation between each type of transcript, mainly genes and isoforms (Fig. 2c, d), we find that variation is highly dependent on transcript expression level; that is, lowly expressed genes and isoforms tend to have higher coefficients of variation than those highly expressed, as previously suggested [22]. However, we still find that aged livers have a higher coefficient of variation, irrespective of expression level. Additionally, we found that the percentage for each category of transcript of total normalized counts is generally more variable for old livers as compared to young (Fig. 2e). These findings indicate that the liver transcriptome from aged mice is consistently more variable than that from young mice and that this increased inter-individual variation is attributable, in part, to the stochastic variation observed at the level of specific transcript types.

### Differential expression of RNA species between young and old mouse liver

Before analyzing individual transcripts we first assessed our data for global changes in expression levels. Since the magnitude of age-related expression differences cannot be predicted and could be small [23], we refrained from setting an arbitrary cut-off for fold change, thus allowing us to detect all possible statistically significant changes in expression. In total we found 1264 transcripts differentially expressed with age in mouse liver. Of these, 974 transcripts were significantly (FDR <0.1) upregulated while 290 transcripts were significantly (FDR <0.1) downregulated in aging (Fig. 1c; Additional file 2: Table S2). Of the total transcripts, we found a total of 1102 significant differentially expressed protein-coding (mRNAs) genes (FDR <0.1; Additional file 2: Table S2). This comprises 863 upregulated genes, and 239 downregulated genes. This finding corroborates previous microarray studies showing that, in general, more genes are upregulated than downregulated in aging liver [5]. As could be predicted, when the top 100 protein-coding transcripts were subjected to unsupervised hierarchical clustering, the old and young mice were separated to each respective cohort (Fig. 1d).

In order to validate results obtained from our RNA-seq dataset we performed qPCR validation by probe-based array. We specifically chose 13 transcripts varying in count number, fold change differences, and as controls genes previously implicated in aging or senescence. For transcripts upregulated in aging liver we analyzed *Ly6a*, *Mmp12*, *Cxcl9*, *Gbp2*, *Il7*, *Rac2*, *Fgfr3*, *Ctss*, and *Terc* (ncRNA), while downregulated transcripts validated were *Mt1*, *E2f7*, *Hspa1b,* and *Neat1* (ncRNA). We directly compared these qPCR results with the RNA-seq results by plotting the linear regression of the log_2_FoldChange(Old/Young) values for each transcript (Additional file 7: Figure S1A). Results showed a Pearson’s correlation coefficient of 0.95, confirming our RNA-seq results are accurate and reliable.

Recently, studies have aimed to discriminate between expressed mRNA isoforms, as mRNA processing could be altered as a function of age [15, 24, 25]. Thus, we tested if we could detect changes in isoform expression in mouse liver by taking our read alignments and utilizing the Cufflinks/Cuffdiff package [26] with the ENSEMBL annotation database. We found a total of 105 differentially expressed isoforms (Additional file 3: Table S3) with age. Of these, 73 isoforms were significantly (FDR <0.1) upregulated, while 32 were significantly (FDR <0.1) downregulated in aged liver. Interestingly, we found isoform switching enriched on chromosome four, where 38 out of the 105 significant isoforms were found. Furthermore, one specific locus, the large MUP (major urinary protein) locus that spans 2.19 Mb at Chr.4qB3, appeared responsible for 33 of these 38 isoforms. These results indicate that mRNA isoform switching is not uncommon during aging of the liver with the MUP locus as a major hotspot.

Several studies have shown potential functional implications for ncRNA changes in both aging and cellular senescence [14, 27–29], most cases involving miRNAs. Utilizing our unique annotation reference database we sought to identify all ncRNAs (i.e. pseudogenes, lncRNAs, snoRNAs, snRNAs, and miRNAs) that were significantly differentially expressed with age in mouse liver. In total we found 162 ncRNAs significantly differentially expressed (FDR <0.1; Additional file 2: Table S2). Of these, those specifically upregulated with age were 56 pseudogenes, 23 lncRNAs, one miRNA, nine snRNAs, one snoRNA, one telomerase RNA component (TERC) and 20 unclassified ncRNAs. Those downregulated were 31 pseudogenes, seven lncRNAs, one miRNA, three snRNAs, and nine unclassified ncRNAs. In keeping with our previous results for global expression changes, more ncRNAs are significantly upregulated, in general, than downregulated with age. Of note, we only find two significant differentially expressed miRNAs (18–22mers) due to the inherent size constraint encountered when sequencing libraries for 100 bp read length on the Illumina platform. To validate these novel ncRNA findings we performed qPCR of a select 13 ncRNA transcripts ranging in type, fold change, and count number. For those up-regulated with age we chose to analyze *Mup-ps4*, *Rian*, *Mup-ps19*, *2410018L13Rik*, *A230056P14Rik*, and *Dleu2* while down-regulated ncRNAs validated were *Gm20577*, *Gm16551*, *Gm2788*, *Gm5911*, *Gm5844*, *Mir17hg*, and *Gm19316* in both young (*n* = 3) and old (*n* = 3) mouse livers. Again, we compared our RNA-seq results to our qPCR results to find that ncRNA expression correlated with a Pearson’s correlation coefficient greater than 0.94 (Additional file 7: Figure S1B), confirming that the novel ncRNAs found in our aging liver are truly differentially expressed.

### Functional annotation

**Fig. 3:**
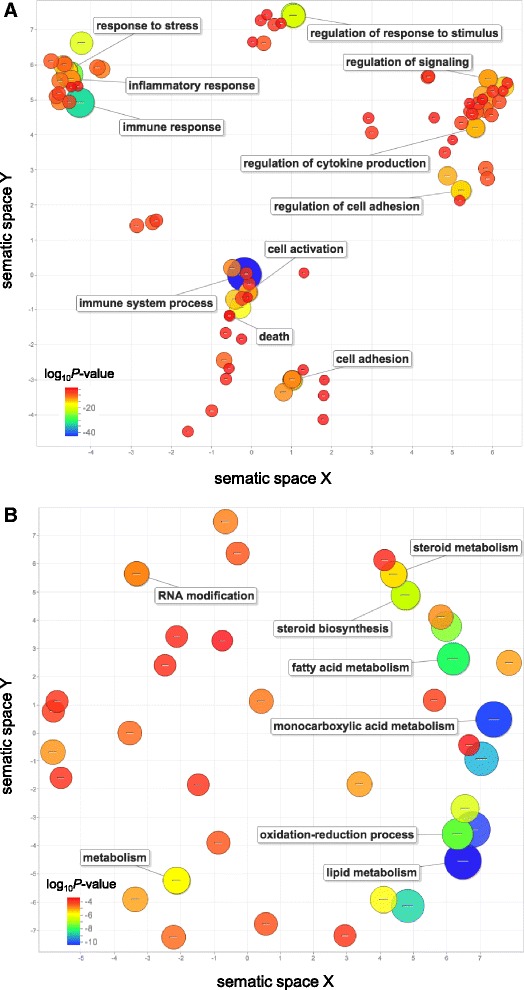
GO term enrichment for genes differentially expressed in aging mouse liver. Enrichment analysis visualized as an MDS plot for GO terms for biological processes that are (**a**) upregulated and (**b**) downregulated in aged mouse liver. Plots are generated based on a matrix of semantic similarities in space (x, y). Clusters of circles closer together represent terms that are more closely related. Circle color and size indicates log_10_ *P*-value

In an attempt to translate the transcriptional landscape in aged mouse liver into functional consequences we first focused on the mRNA component of observed alterations and using both DAVID, the Database for Annotation, Visualization, and Integrated Discovery [30] and GOrilla [31], to perform separate functional annotation analysis on significantly upregulated and downregulated transcripts. In total, 340 gene ontology (GO) annotations were significantly (FDR <0.01) upregulated (Additional file 4: Table S4) while only 51 were downregulated (Additional file 5: Table S5). We then used REVIGO, which uses utilizes semantic similarity to parse GO annotations in order to remove redundant terms and to visualize clusters of enriched terms [32]. GO annotations that were significantly upregulated in aging liver were highly enriched for immune system processes, immune response, response to stress, cell activation, regulation of cytokine production, and cell death (Fig. 3a). GO annotations that were significantly downregulated with aging, were enriched for lipid metabolism, oxidation-reduction process, monocarboxylic acid metabolism, fatty acid metabolism, steroid biosynthesis, and RNA modification (Fig. 3b). Together these findings show that aging liver is highly enriched for an increase in immune response and inflammation as well as a decrease in metabolic annotations, as previously reported [5, 23], while also revealing new clusters such as regulation of cytokine production and RNA modification.

We also performed gene ontology analysis on significant differentially expressed isoforms (Table 1), again either up or downregulated. Here we found that the corresponding genes for isoforms that were upregulated resulted in eight processes highly enriched for immune system function, most notably immune response (GO:0006955), while genes for downregulated isoform analysis gave one significant biological process, oxidation-reduction (GO:0055114). Hence, the isoform pathway analysis corroborated our findings with the gene ontology analysis.

**Table 1:** GO analysis from DAVID for genes from differentially expressed aging isoforms

GO term	Gene count	*P*-value	FDR-value
Upregulated			
Immune response	7	1.7 × 10^−4^	4.7 × 10^−2^
Antigen processing and presentation of exogenous peptide antigen via MHC class II	3	3.8 × 10^−4^	5.3 × 10^−2^
Antigen processing and presentation of peptide antigen via MHC class II	3	3.8 × 10^−4^	5.3 × 10^−2^
Antigen processing and presentation of peptide or polysaccharide antigen via MHC class II	3	5.5 × 10^−4^	5.1 × 10^−2^
Oxidation reduction	7	1.1 × 10^−3^	6.3 × 10^−2^
Antigen processing and presentation of exogenous antigen	3	1.2 × 10^−3^	5.5 × 10^−2^
Antigen processing and presentation peptide antigen	3	1.9 × 10^−3^	3.3 × 10^−2^
Downregulated			
Oxidation reduction	7	3.1 × 10^−4^	5.5 × 10^−2^

We also found many differentially expressed ncRNAs that may play roles in the molecular mechanisms of aging. The first is that of *Neat1*, a lncRNA downregulated in our dataset, which has been associated with nuclear paraspeckle formation [33], as well as nuclear retention of mRNAs [34]. Loss of *Neat1* may well be one explanation for the increase in transcriptional noise that we have previously characterized and possibly that of decreased nuclear organizational structure. We also observed a ~2.5 fold increase in *Pvt1*, a ncRNA inducible by *p53* [35]. *Pvt1* is part of a syntenic locus which is a known hotspot for multiple cancers as well as a host for seven annotated miRNAs [36] that have been shown to decrease expression of *Myc*. Strikingly, *Myc* is down-regulated ~1.93 fold in our dataset (*P* = 0.0036, FDR = 0.127), albeit it barely missed our cut-off of FDR <0.1.

Finally, we analyzed our ncRNA dataset for possible spatial relationships within the genome. Indeed, we observed three ncRNAs, all upregulated in aged liver, on mouse Chr.12qF1 (Additional file 8: Figure S2), which are adjacent to each other. These ncRNAs, *Meg3*, *Rian (Meg8)*, and *Mirg* are a unique set of lncRNAs that have been identified as part of the imprinted locus *Dlk1-Dio3* and are expressed from the maternal allele [37]. Together these lncRNAs cooperate to actively regulate cell proliferation through gene expression either by directly binding and recruiting the polycomb repressive complex two (Prc2) [38], as is the case with *Meg3* and *Rian*, or by predicted microRNA-mediated regulation of cell cycle factors such as *Myc* and *p53* [37], as is the case with *Mirg*. We also found two significantly upregulated novel lncRNAs, *Gm12602* and *Gm12648*, which flank the *Cdkn2a* (*p16*^*ink4a*^ and *p19*^*Arf*^) locus on mouse Chr.4q. Although much less is known about these particular transcripts, *Gm12602* is located directly downstream (~150 kb) and *Gm12648* upstream (~4.5 Mb) of *Cdkn2a*. This makes it tempting to speculate that these lncRNAs may exert some regulatory control of this locus, possibly contributing to senescent phenotypes. Together, these findings point towards a host of age-related ncRNAs as regulators of aging pathways and networks.

### Interaction network analysis

**Fig. 4:**
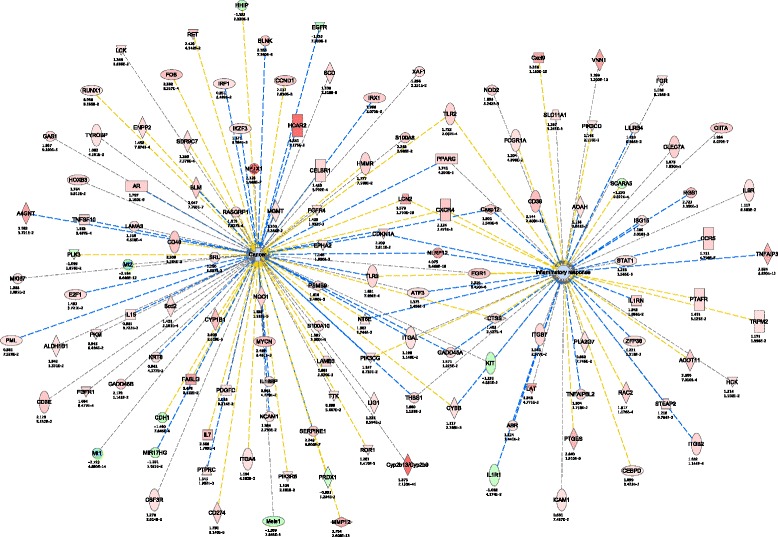
Age-related interaction network for cancer and inflammatory response. Each gene is colored according to directionality of expression; red=upregulated, green=downregulated. Numbers below each gene represent the fold change (top) and FDR value (bottom) of that particular gene. Orange line=predicted activation, blue line=predicted inhibition, yellow line=prediction inconsistent, grey line=no predicted effect

**Fig. 5:**
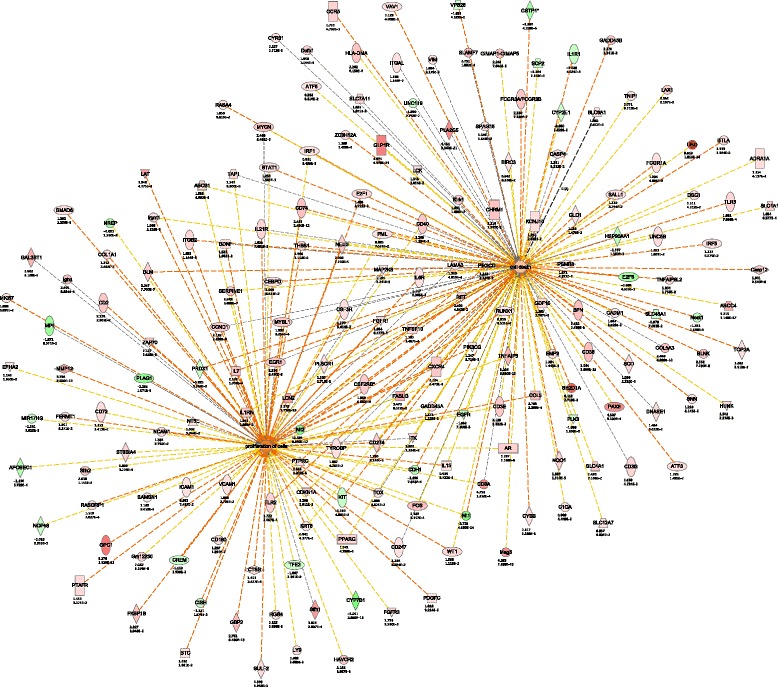
Age-related interaction network for proliferation of cells and cell death. Each gene is colored according to directionality of expression; red=upregulated, green=downregulated. Numbers below eachgene represent the fold change (top) and FDR value (bottom) of that particular gene. Orange line=predicted activation, blue line=predicted inhibition, yellow line=prediction inconsistent, grey line=no predicted effect

**Fig. 6:**
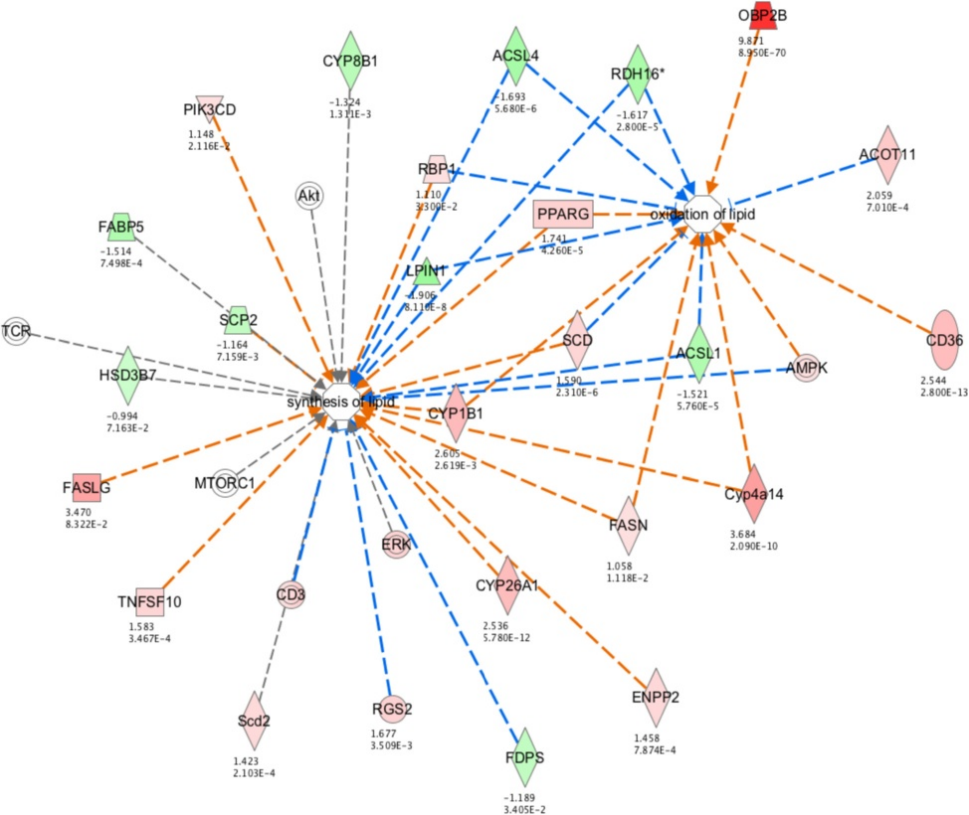
Age-related interaction network for synthesis of lipid and oxidation of lipid. Each gene is colored according to directionality of expression; red=upregulated, green=downregulated. Numbers below each gene represent the fold change (top) and FDR value (bottom) of that particular gene. Orange line=predicted activation, blue line=predicted inhibition, yellow line=prediction inconsistent, grey line=no predicted effect

The increased accuracy and breadth of our RNA-seq data sets allowed us to generate networks of gene functional change in aging liver, above and beyond what was observed using DAVID or GOrilla. Using Ingenuity Pathway Analysis (IPA) we generated, from the differentially expressed protein-coding genes and ncRNAs, interaction networks of functional change. This resulted in multiple overlapping pro-aging networks from which we could distinguish three major molecular phenotypes: inflammation, proliferative homeostasis and lipid metabolism (Figs. 4, 5 and 6).

Of the multiple networks of liver aging, the most prominent interaction network to emerge was inflammation and cancer (Fig. 4). This was not surprising since this could already be derived from the gene ontology analysis and has also been previously reported [5, 16]. This is in keeping with observations that inflammatory cytokines secreted from senescent cells are capable of promoting the hyperplastic growth of surrounding cells [39, 40]. We, and others, previously observed an increase of senescent cells in aging liver [16, 41, 42], which could promote the age-related increase of cancer through these same secreted cytokines. Conversely, it has also been shown that infiltrating immune cells can clear pre-oncogenic senescent hepatocytes [43], thereby the same secretory phenotype emitted by cells to promote cancer can also act a barrier to tumorigenesis in the liver.

Another prominent age-related network that emerged from the IPA analysis was that of proliferation of cells and cell death. This points to an age-related loss of proliferative homeostasis, which is in keeping with reports suggesting that aging compromises the rate of liver regeneration rather than its regenerative capacity [44]. These two connected but opposing networks (Fig. 5) link together genes shown to be highly important in aging such as *Cdkn1a*, *Il6R*, *Mmp12*, *Top2a*, *Pdgfc*, *Lama3*, and *E2f1* [45–47]. Considering the liver is a still a mitotically active tissue, this interplay emphasizes the complex interactions needed to maintain its normal regenerative function. An aging phenotype to arise from the breakdown of cellular proliferation is the increase in cellular senescence. However, IPA does not contain a formal pathway for cellular senescence per se, which is essentially a stress response and varies based on the senescent trigger, i.e. genotoxic stress induced, oncogene induced, or replication induced senescence. Of note, many of the genes involved in the cancer network were also implicated in the proliferation of cells network, as could be expected since cancer is the uncontrolled proliferations of cells.

The third major interacting network of age-related change, which is the interaction between the synthesis of lipids and the oxidation of lipids (Fig. 6), is enriched for genes that are downregulated in aging. Clearly, the most abundant enzyme family involved in this network is the cytochrome P450 family (*Cyp8b1*, *Cyp1b1*, *Cyp4a14*, *Cyp26a1*). While there certainly is evidence for reduced hepatic drug clearance in humans and rodents, the possibility that this is caused by reduced P450 activity is controversial [44]. One phenotypic outcome arising from the failure of these pathways to cooperate could be the increase in one of the most prevalent pathological outcomes in liver, lipofuscin accumulation [48]. This phenotype could reflect the inability to eliminate cellular waste products. A decline in liver metabolic activity with the subsequent increase in lipofuscin with age could be due in part to the decrease in chaperone mediated autophagy [49].

Since protein-protein interactions are very well studied as compared to those of ncRNAs, by analyzing both protein-coding and ncRNAs together, the most robust networks resulting from this analysis would only contain protein-protein interactions, essentially losing the weaker, less-studied ncRNA interactions. Thus, we chose to separately analyze only ncRNAs with age-altered expression to create age-related ncRNA interaction networks. Subsequently, we looked at the proteins and genes listed in our ncRNA interaction network and filtered the list for those only differentially expressed within our dataset.

**Fig. 7:**
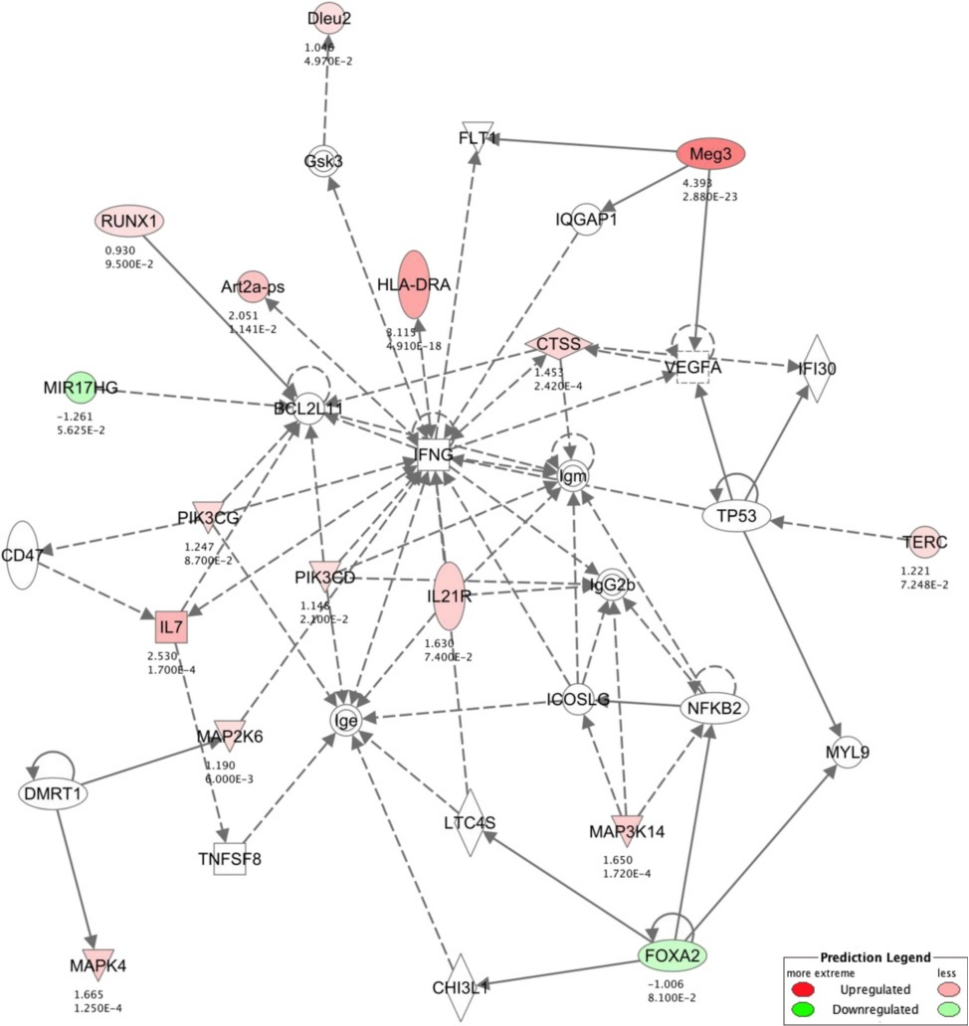
NcRNAs associate in INFG-mediated pro-inflammatory network. NcRNA network analysis reveals functional role for non-canonical transcript involvement in *INFG-*mediated pro-inflammatory response. Colored nodes represent directionality of transcript expression within dataset; *red*?=?upregulated, *green*?=?downregulated, *white*?=?no significant change. Numbers below each gene represent the log_2_FoldChange (*top*) and FDR value (*bottom*) of that particular gene

Only four interacting networks were obtained in this way, three of which only had one ncRNA interacting with only one other protein or gene, similar to previous ncRNA network analysis [29]. Only one network had multiple known interactions within the IPA database; thus, we focused on this network that included five ncRNAs with known interactions to other proteins and/or genes. By combining the ncRNAs and the known interacting protein-coding genes altered with age and again analyzing with IPA we were able to create an age-related ncRNA-protein coding interaction network. This one robust network association (Fig. 7) is involved in the *IFNG*-mediated pro-inflammatory response. Interestingly, this network links our novel ncRNAs to certain genes such as *p53*, *Il7*, *Ctss*, and *NFκB*, all of which have been previously associated with mammalian aging or senescence [23, 50, 51], and their age-related alterations may contribute to the most prevalent aging phenotype, inflammation.

## Discussion

Functional and pathological changes in liver with age have been studied extensively, but the extent of physiological decline of this organ over time remains controversial [52, 53]. Hence, it is critically important to comprehensively assess changes at the molecular level, which could at least in part resolve this issue. Next-generation sequencing technology has opened up the possibility to study the molecular basis of aging in unprecedented detail. In studying gene expression, generally thought to be the most immediate reflection of critical, age-related alterations in any tissue-specific phenotype, the advent of RNA sequencing now allows a full depiction of the aging transcriptome. As we show here for mouse liver, the increase in experimental detail as compared to previously applied microarrays is dramatic. While previous, microarray-based studies revealed highly variable numbers of differentially altered genes during aging [8, 9], and sometimes none at all [6], our RNA-seq data sets showed a total of 1102 protein-coding transcripts as differentially expressed between livers of young and old mice, plus an additional 105 differentially expressed isoforms and 162 differentially expressed ncRNAs. The latter is undoubtedly an underestimate due to the limited number of such RNAs annotated thus far. Albeit limited to two extreme age levels and one mouse strain, the current scope of our study highlights the complexity of transcriptome changes in aging mammals. Indeed, such complexity is likely higher still when possible strain differences and variation across multiple age levels will be taken into account. However, such unprecedented detail in visualizing the aging transcriptome allows us not only to uncover the major gene networks of phenotypic change with age, but also to test some very basic hypotheses about loss of transcriptional fidelity as a possible ultimate cause of aging.

One obvious characteristic of the aging transcriptome in liver is the increased individual variation. Evidence for the importance of stochastic components in gene expression changes with age has been provided previously using different approaches [20, 54], but ours is the first direct evidence that transcriptomes of individual animals can significantly diverge as a consequence of the aging process, and not just between individual genes. Indeed, as we show, aging is accompanied by an increase in stochastic variation, or transcriptional noise, which is apparent in all RNA classes. Our finding that older animals, in general, have higher coefficients of variation, irrespective of transcript length, confirms that age-related variation is genome-wide. However, we do observe that lowly expressed transcripts, irrespective of age, have much higher coefficients of variation as compared to highly abundant transcripts. This could be due to the inherent labile nature of smaller RNA species, as longer highly expressed transcripts tend to be more stable and less susceptible to metabolism, either due to higher order structure or because more proteins are able to bind and protect the transcript from endogenous RNases [55, 56]. In this respect, it is striking that we find RNA modification as an enriched pathway that is downregulated within our dataset, a possible reason that aged transcriptomes tend to have higher variation. Moreover, the downregulation of *Neat1* may contribute to variation between mRNAs containing inverted Alu repeats in the 3′ UTR [34]. Thus, the possibility cannot be ruled out that specific pathways ultimately control stochastic variation in the aging transcriptome, possibly as part of a systemic response.

One longstanding hypothesis in gene expression is that as an organism ages, the genome loses its ability to effectively regulate genes, leading to an overall relaxation or de-repression of gene expression [19]. Our data do not confirm the widespread occurrence of such a loss of gene transcriptional control, as we did not observe a drastic increase in genomic coverage of the transcriptome from young to old animals. However, to definitively rule out this hypothesis, extremely deep sequencing, i.e., greater than 1 billion reads, may be necessary [18]. Alternatively, reports on the de-repression of gene expression have been limited to a select few tissues and therefore the observation could be a function of tissue or cell type specificity.

A major challenge in attempts to fully characterize the transcriptional landscape at old age is linking altered mRNA expression levels to altered ncRNAs, recently highlighted as a key regulatory component of the transcriptome and in aging [14, 57]. Our results show that roughly 13 % of the transcripts we found altered with age are those of ncRNAs. Although many of these ncRNAs have been previously reported and studied, such as the lncRNAs *Meg3*, *Rian*, and *Neat1,* none of these to our knowledge have been implicated in aging. Additionally, it is noted that the *Dlk-Dio3* locus in mouse corresponds to the largest hotspot of miRNAs within its genome. Interestingly, a previous report has shown that eight miRNAs originating within this cluster are downregulated in aged skeletal muscle [29]. Coupled with our findings that lncRNAs (*Meg3, Rian, Mirg*) from this locus are upregulated in aging liver, we can conclusively define this locus as cell-type specific regulatory hotspot in aging. Whether this locus plays a major regulatory role in the aging process remains to be elucidated, but could be studied by testing if multiple proteins and/or pathways which are targeted are actually affected in aging tissues. In addition to known ncRNAs, we also revealed novel lncRNA transcripts differentially expressed with age, such as those flanking the *Cdkn2a* locus. Given the importance of the *Cdkn2a* locus in both senescence and cancer, these flanking lncRNAs may offer putative targets to regulate the cell cycle. More evidence for the implications of such ncRNAs in aging will become more apparent as the field expands to functionally characterize each novel ncRNA. Taken together, our results provide the first evidence that ncRNAs can also shape the landscape of aging mouse liver.

Finally, the comprehensive view of the aging liver transcriptome provided in our present study allowed us to determine the main functional networks associated with liver aging. In this respect our results uncovered three main sets of interacting networks: inflammation and cancer, proliferative homeostasis, and lipid metabolism. The most prominent one, inflammatory response, was also supported by multiple ncRNAs novel to liver aging in the *INFG*-mediated pro –inflammatory response. Increased inflammation has since long been associated with aging [58] and we and others have previously provided evidence that this is a major pro-aging phenotype in the mouse [59]. A second major set of altered gene functional networks in aging mouse liver involves the interconnected cellular proliferation and death networks. Systematic dysregulation of the balance between degenerative cell loss and regeneration is in keeping with reports suggesting that liver regeneration is compromised in old animals and in elderly humans. Lastly, a third major network of change appeared to be centered on lipid synthesis and lipid oxidation, pointing towards increased intracellular aggregates of damage, most notably lipofuscin. However, our data also suggest altered levels of cytochrome P450 family, such dysregulation could be a cause for the decline in drug metabolism in aging livers [53].

## Conclusions

Taken together, our present work, and previous work by others [14, 15], shows how RNA sequencing at high depth can address basic questions as to the molecular basis of mammalian aging. We showed that aging is unlikely to be accompanied by a significant loss of tissue-specific gene expression profiles; we did not find evidence for gene de-repression or a stochastic increase of the fraction of the genome that is transcribed. Yet, our data uncovered extensive changes in gene expression patterns, which are subject to stochastic variation as indicated by a significant increase in individual variation. We also demonstrated that the increased accuracy of RNA-seq enable us to better capture major aging phenotypes at the transcriptional level, thereby unmasking important molecular mechanisms underlying aging and its related disease sequelae.

## Methods

### Animals and tissue collection

All procedures involving animals were approved by the Institutional Animal Care and Use Committee (IACUC) of Albert Einstein College of Medicine. Three male Balb/C mice of 4 and 28 months of age were procured from the National Institutes on Aging. Mice were sacrificed and harvested liver samples were immediately flash-frozen. All mice used in this study were determined to be tumor-free and also lacked other obvious signs of macropathology at the time of sacrifice.

### RNA-seq

Directional RNA sequencing libraries and raw sequencing data were previously generated by us [16].

### Analysis and statistics

Pass filter sequences were aligned using GSNAP v2013-01-23 according to default settings with novel splicing, using the NCBI Build 37/mm9 reference genome [60]. Alignments were then referenced against a custom annotation database that combines ENSEMBL (release 71), GENBANK/NCBI (release 196); REFSEQ (release 60), VEGA/HAVANA (release 51); and MGI (GRCm38). The custom annotation database is available upon request. Uniquely aligned reads were then quantified using Python based HTSeq for paired-end reads (for fragment based quantification) using the intersection-strict model and subsequently analyzed using the DESeq package in R [61]. For isoform analysis, aligned reads were quantified using the Cufflinks/CuffDiff package for paired-end settings with the Ensembl database and subsequently analyzed using CummeRbund in R [26]. For all analyses, a *P*-value <0.05 was considered significant and all *P*-values were adjusted for multiple testing using Benjamini-Hochberg correction where a cutoff FDR value <0.1 was considered significant.

### Gene ontology and network analysis

GO analysis was performed using either DAVID analytical software for GOTERM_BP_FAT by using an EASE score <0.05 and gene set count minimum of two required for a functional pathway to be considered significant [30] or GOrilla where four genes were required to be considered in each annotation and a q-value cutoff <0.01 [31]. Gene ontology enrichment was visualized using REVIGO with a similarity cutoff set at 0.7 [32]. For network analysis, differential gene expression lists were analyzed using Ingenuity Pathway Analysis (Ingenuity® Systems, www.ingenuity.com). IPA also was used to generate figures for network interaction of gene sets for ncRNAs. Data visualization was performed in the UCSC genome browser [62].

### qPCR

Total RNA from young and old mouse liver was converted into cDNA using SuperScript III First-strand Synthesis Kit (Invitrogen) using 50 ng of random hexamers. qPCR was performed using 40 ng of cDNA using ABI StepOne Plus system for either TaqMan® (ABI) or SYBR® green (ABI) assays. For a full list of Taqman primer assays and custom non-coding primer assays used see Additional file 6: Table S6. All calculations were performed using the ΔΔCT method with TaqMan assays normalized to eukaryotic 18 s rRNA and SYBR assays normalized to GAPDH with all biological replicate values representing the mean of technical triplicates. Linear regression was used to calculate the Pearson’s correlation coefficient of the log_2_foldchange values between RNA-seq and qPCR.

## Data availability

Raw RNA-seq data from this manuscript were deposited to the Sequence Read Archive under accession code SRP053350. The custom annotation database made in this study for mouse is freely available on GitHub (https://github.com/rrwhite15/mouse-annotation-gtf-file).

## Additional files

Additional file 1: Table S1. Sequencing and alignment metrics for each young and old liver sample. (XLSX 44 kb) Additional file 2: Table S2. Expression level, fold change, and biotype of each significantly differentially expressed transcript between young and old livers. (XLSX 287 kb) Additional file 3: Table S3. List of top differentially expressed isoforms between young and old livers. (XLSX 73 kb) Additional file 4: Table S4. List of top GO terms from upregulated differentially expressed genes used to make Fig. 3a. (XLSX 198 kb) Additional file 5: Table S5. List of top GO terms from downregulated differentially expressed genes used to make Fig. 3b. (XLSX 60 kb) Additional file 6: Table S6. List of primer sets used in RNAseq validation. Custom ncRNA primers are provided with a forward and reverse primer while TaqMan Assay ID is given for all others. (XLSX 52 kb) Additional file 7: Figure S1. RNAseq validation. (a) qPCR validation of 13 selected transcripts normalized to eukaryotic 18 s rRNA using TaqMan primer/probe sets. (b) qPCR validation of 13 differentially expressed non-coding RNAs normalized to *GAPDH* mRNA using custom made primer for each novel ncRNA. For both, RNA-seq and qPCR values are plotted as the mean (Old, *n* = 3; Young, *n* = 3) log_2_FoldChange(Old/Young) value, where the red line indicates the slope of linear regression and the square of Pearson’s correlation coefficient (*r*). Insets are bar plots of equivalent corresponding values. (PDF 122 kb) Additional file 8: Figure S2. Representative subset (*Meg3*, *Rian* and *Mirg*) of maternally expressed and imprinted ncRNA locus, *Dlk-Dio3*, on chromosome 12 in UCSC Genome Browser overlaid with aligned reads from RNA-seq dataset. Blue = sense paired-end, red = antisense paired-end; Scale = 100 kb. (PDF 64 kb)
