# Supplementary figures and images for: Comprehensive transcriptional landscape of aging mouse liver
Source: BMC Genomics. 2015 Nov 5;16:899. doi: 10.1186/s12864-015-2061-8 (PMC4636074; doi:10.1186/s12864-015-2061-8)

Supplemental Figure S1

A

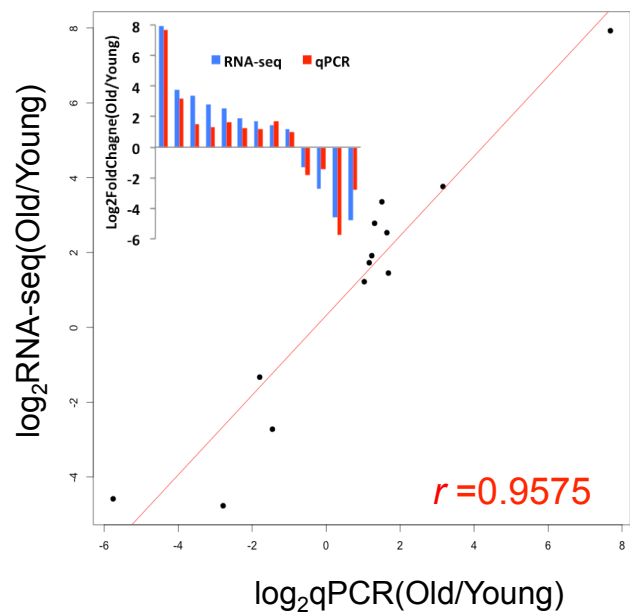

B

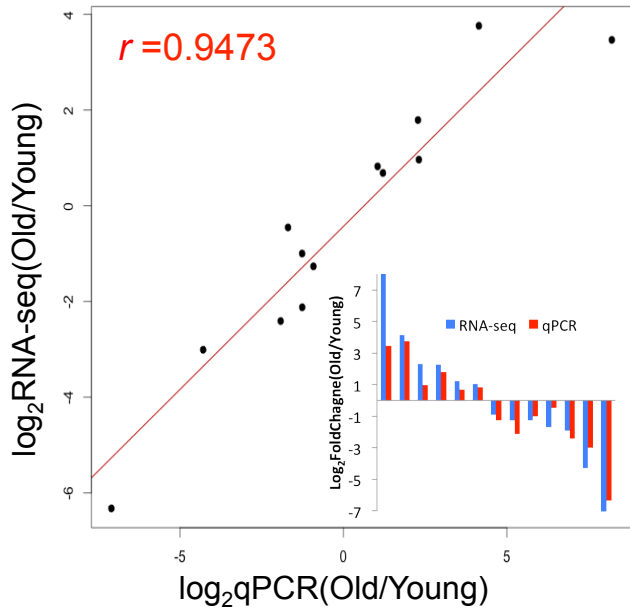

Supplement: Additional file 7: Figure S1. — RNAseq validation. (a) qPCR validation of 13 selected transcripts normalized to eukaryotic 18 s rRNA using TaqMan primer/probe sets. (b) qPCR validation of 13 differentially expressed non-coding RNAs normalized to GAPDH mRNA using custom made primer for each novel ncRNA. For both, RNA-seq and qPCR values are plotted as the mean (Old, n = 3; Young, n = 3) log2FoldChange(Old/Young) value, where the red line indicates the slope of linear regression and the square of Pearson’s correlation coefficient (r). Insets are bar plots of equivalent corresponding values. (PDF 122 kb) [file 12864_2015_2061_MOESM7_ESM.pdf]
