# Supplementary material for: Comprehensive transcriptional landscape of aging mouse liver
Source: BMC Genomics. 2015 Nov 5;16:899. doi: 10.1186/s12864-015-2061-8 (PMC4636074; doi:10.1186/s12864-015-2061-8)

Supplemental Figure S2

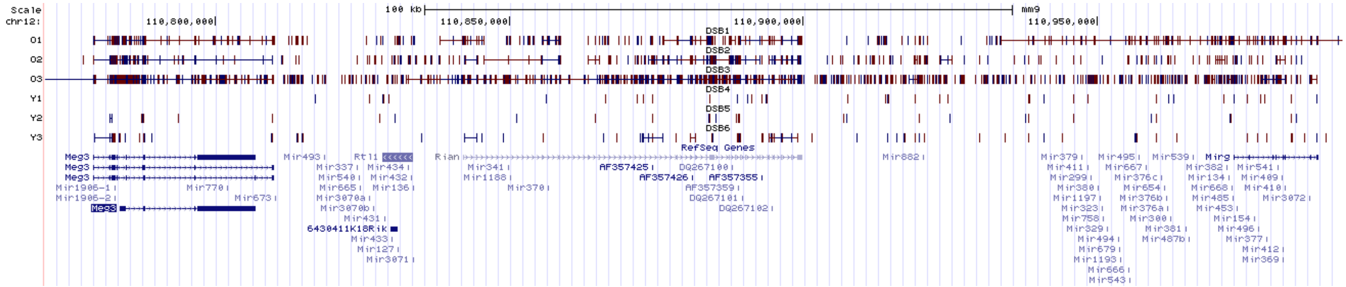

Supplement: Additional file 8: Figure S2. — Representative subset (Meg3, Rian and Mirg) of maternally expressed and imprinted ncRNA locus, Dlk-Dio3, on chromosome 12 in UCSC Genome Browser overlaid with aligned reads from RNA-seq dataset. Blue = sense paired-end, red = antisense paired-end; Scale = 100 kb. (PDF 64 kb) [file 12864_2015_2061_MOESM8_ESM.pdf]
